# Single-port robotic surgery in gynecology: first experience in German-speaking countries using the Da Vinci SP system in benign andneoplastic diseases

**DOI:** 10.1007/s11701-025-02983-1

**Published:** 2025-12-01

**Authors:** Zaher Alwafai, Emma Schweder, Broder Poschkamp, Melanie Langheinrich, Stephan Kersting, Richard Hummel, Marek Zygmunt

**Affiliations:** 1 Department of Obstetrics and Gynecology, University of Greifswald, Greifswald, Germany; 2 Department of Ophthalmology, University of Greifswald, Greifswald, Germany; 3 Department of General, Visceral, Thoracic, and Vascular Surgery, University of Greifswald, Greifswald, Germany

**Keywords:** Robotic surgery, Minimally invasive surgery, Single-port system, Da vinci SP

## Abstract

**Introduction:**

The Single-Port (SP) surgical system represents a significant advancement in robotic surgery, facilitating procedures through a single incision while overcoming the previously reported limitations of single-site surgery. As the first clinic in the German-speaking countries to use the da Vinci SP system for gynecological surgeries to treat benign and malignant diseases, we aim in this study to report our initial experiences and outcomes.

**Patients and methods:**

This retrospective study included 62 patients who underwent gynecological surgeries using the da Vinci SP system. Various procedures were performed, patients diagnosed with both benign and malignant conditions were included in the study.

**Results:**

We performed hysterectomy (*n* = 31); hysterectomy with pectopexy (*n* = 6); pectopexy alone (*n* = 7); salpingo-oophorectomy (*n* = 12); myomectomy (*n* = 1); and endometriosis surgery (*n* = 5). The mean operation time was 136.2 ± 70.3 min. The mean docking time was 8.3 ± 2.2 min (range, 3–13). The mean hemoglobin level changed significantly by -0.93 ± 0.61 mmol/L (-1.53 ± 0.98 g/dL) (paired t-test, *p* < 0.001). No patients required blood transfusion. Notably, no patients developed incisional hernias following the operation. Two patients had minor postoperative complications. The mean hospital stay was 3.6 days ± 1.7 days with operative time as associative factor (+ 0.71 days per operative hour, *p* = 0.044).

**Conclusion:**

Our initial experience demonstrates promising results with the use of the da Vinci SP system in gynecology. Single-port robotic surgery seems to be safe and offers several advantages over traditional multi-port systems, with the potential to complement existing platforms. However, it entails a new learning curve for surgeons.

## Introduction

### Robotic surgery

Minimally invasive surgery has revolutionized surgical practice in gynecology by offering multiple advantages due to less tissue trauma and reduced blood loss. This results in less postoperative complications, reduced length of hospital stay, faster return to daily activities, and improved cosmetic outcomes for patients have been reported [1–4]. Robotic-assisted surgery is an innovative technology for performing minimally invasive surgery. It offers enhanced precision, improved visualization, and better ergonomics for surgeons [5, 6]. After initial applications of robotic-assisted surgery on humans during the 1980s, Intuitive Surgical Inc. (Sunnyvale, CA, USA) was founded in 1995. Subsequently, in 2000, the Food and Drug Administration (FDA) approved the first da Vinci surgical system for robotic-assisted surgery [7, 8]. CE-mark approval had already been granted in Europe in 1999 [9]. Robotic surgery has gained widespread acknowledgment and popularity the last years and the number of surgeons performing robotic assisted procedures worldwide have consequently increased. Since 2005, gynecologists in the USA are officially allowed to use the da Vinci surgical system for gynecological indications [10]. The da Vinci surgical system is equipped with a high-definition camera that has 3D view. This improved visualization is beneficial for challenging surgical cases [11]. The system can furthermore reach a higher degree of freedom in motion with its wrist-like robotic instruments, which can be controlled through a remote console by qualified surgeons. These features make it possible to operate with more efficiency and precision, especially in tight operating spaces, like the pelvic cavity [6, 12].

### Single-port surgery

For even better cosmetic outcome and improved patient satisfaction, single-port surgery was invented. Initially, surgeons performed single-port laparoscopies, but this technique brought forth new challenges, such as poor visualization, limited movement and collisions of instruments [13]. Later, as robotics gained popularity in gynecological surgery, a combination of single-port surgery and robotic surgery was developed: single-site robotic platforms. The da Vinci Single-Site platform was designed to enable surgeons to perform surgery through a single incision using a multiport robotic system [14]. This improved ergonomics but still came with issues, like instrument crowding and clashing. Single-Site platform was equipped with semi-rigid instruments, which were less versatile and powerful [15]. To overcome the challenges of robotic single-site surgery, Intuitive Surgical (Sunnyvale, CA, USA) introduced the da Vinci Single-Port (SP) surgical system, which was approved for urological surgeries in 2018 and for otolaryngology procedures in 2019 [16–19]. To date, it has not been approved for gynecological procedures in the USA [16]. In Europe, the system received its CE-mark approval in 2024 [9]. The da Vinci SP surgical system uses high flexible EndoWrist SP instruments and a camera with COBRA action [20], which allows flexible, snake-like movements for enhanced visualization in confined spaces.

### Aim of study

Few previous, mainly Asian, studies, have reported the initial experiences with the da Vinci SP in gynecological procedures, demonstrating non-inferiority compared to the multiport systems. A reduced docking time and less blood loss compared to multiport surgery are some of the benefits provided by operating with the da Vinci SP surgical system [21–24].

Since December 2024, we have been the first institution in the German-speaking countries (Germany, Switzerland, and Austria), and among the first in Europe, to utilize the da Vinci SP system for gynecological surgery. This study aims to report our initial experience with the system in the management of both benign and malignant gynecologic conditions. By sharing experience, we intend to help other clinicians in adopting the SP platform, facilitate the learning process, and provide an overview of its advantages, limitations, and potential areas for further development.

## Patients and methods

We conducted a retrospective, single-center analysis to describe our initial experience with the da Vinci SP System in gynecological procedures. Our objective was to evaluate the outcome, to compare it with findings reported in the literature, and to find practical issues related to SP surgery. The present study was approved by the local ethics committee of the university hospital of Greifswald (BB 146/25).

### Patients and data collection

This study included 62 patients who underwent gynecological surgeries using the da Vinci SP system between December 2024 and September 2025 at the Department of Obstetrics and Gynecology, University Hospital Greifswald in Germany. Various procedures were performed, including benign and oncologic hysterectomies with or without lymph node dissection, pectopexy, salpingo-oophorectomy, myomectomy, and endometriosis surgery. All procedures were carried out by two qualified surgeons. Patients were selected randomly according to the operative capacity of our clinic, without specific inclusion or exclusion criteria, as this was a retrospective study.

Patient data and related clinical information were retrospectively collected using the patients’ medical records in the hospital data system. Data regarding patients’ characteristics (age, body mass index (BMI), comorbidities, and factors potentially affecting surgical outcomes), intra- and postoperative complications, conversions to laparotomy, pre- and postoperative hemoglobin changes, postoperative pain, total operation time, docking time, and length of hospital stay were included. The operation notes were used for collecting intraoperative information. The total operating time was defined as the duration between the initial surgical procedure and the skin closure. When available, we also documented the docking time, defined as the duration from the initial abdominal skin incision to the insertion of all instruments through the access port, correctly positioned intraabdominally and ready for use via the console. Preoperative and postoperative hemoglobin levels were measured at patient admission and during the hospital stay (mmol/L). Postoperative pain scores were recorded by the ward nursing staff using the Numeric Rating Scale (NRS), a simple and widely used tool for assessing pain intensity at rest and during physical activity. The NRS ranges from 0 to 10, with 0 indicating no pain and 10 representing the worst possible pain. Pain assessments were performed on the first and on the second to third postoperative days. The postoperative analgesia and dosing were administered according to clinic standards: Paracetamol, up to 1 g four times daily, and Ibuprofen, up to 600 mg three times daily. Complications and hospitalization times were collected during the daily bedside visits and discharge examinations.

### SP system

**Fig. 1:**
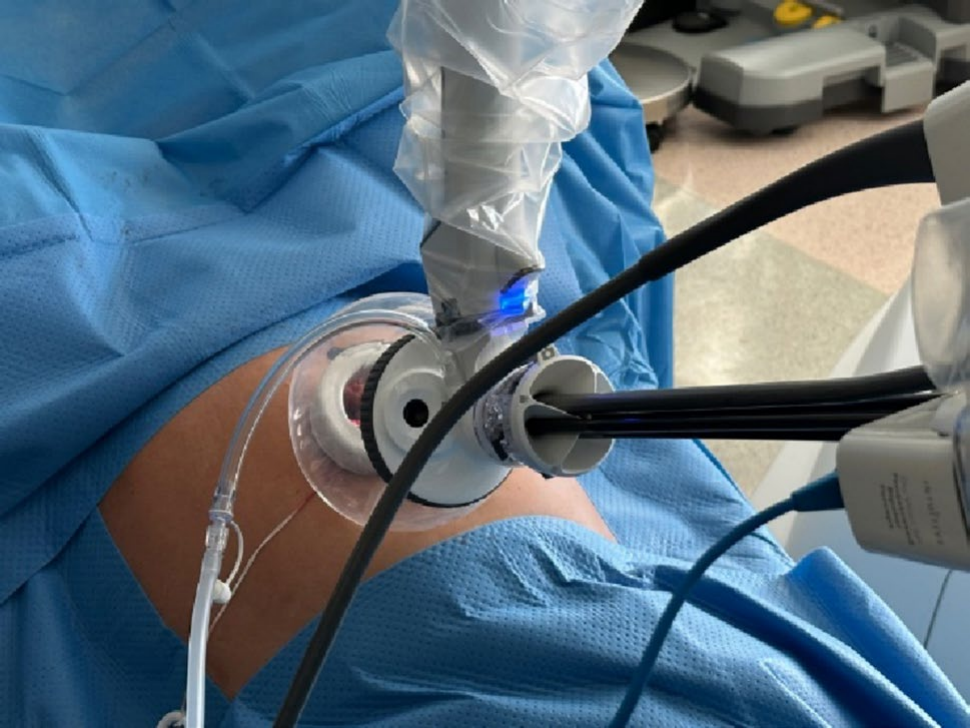
A 6 cm diameter access port was inserted through the umbilicus, and a 0-degree camera along with three instruments were introduced during the operation

The SP-System made it possible to perform the whole operation through a small single incision with just one port. The positioning of the instruments occured through a 25-mm SP multichannel canula or an access port (Fig. 1.). The instrument arm was equipped with four instrument drives that can handle one camera and up to three instruments simultaneously. The 12-mm oval EndoWrist SP camera, which had a 73-degree field of view, also helped with improved visualization. All instruments have a wrist and elbow joint, allowing them to achieve high flexibility and precision [2, 5, 11].

The SP system features an advanced instrument guidance system displayed on the monitor during surgery. It provides a 3D view that simulates the position of all instruments, including the camera, enabling better orientation of the instruments within the surgical field.

### Surgical procedures

A 2.5–2.7 cm vertical or horizontal periumbilical incision was made to facilitate insertion of the access port into the abdominal cavity, following a layer-by-layer dissection of the abdominal wall. This incision size is the minimum required for the SP system. After placement of the da Vinci SP access port, pneumoperitoneum was established using carbon dioxide insufflation to a pressure of 12 mmHg. A 3D endoscope was then introduced through the access port, and a thorough inspection of the abdominal cavity was performed. Subsequently, the patient was placed in the Trendelenburg position, with the head tilted approximately 20–25 degrees downward. Three EndoWrist SP instruments, selected at the discretion of the operating surgeon, were then inserted. An additional 0.5 cm incision was made in the lower abdomen to accommodate an assistant trocar in some cases.

The surgical steps for each procedure were performed in accordance with the standards of minimally invasive gynecological surgery. The instruments utilized during the procedures included fenestrated bipolar forceps, monopolar curved scissors, Cadiere forceps, and needle drivers. Upon completion of the planned surgical intervention, all instruments were withdrawn under direct visualization. The robotic system was disconnected, and the access port was removed. Fascial closure was achieved using a continuous suture, while skin closure was performed with interrupted suture. All patients received a prophylactic preoperative broad-spectrum antibiotic, and no postoperative antibiotics were required. All surgeries were performed by two experienced oncological surgeons certified in robotic surgery, each with extensive experience in multiport robotic procedures, and who had completed training in single-port robotic surgery.

### Statistical analysis

Data cleaning and formatting were performed using Python 3.10.18 (Python Software Foundation) and Microsoft Excel. Statistical analysis and visualization were conducted with Python and Prism (GraphPad v10.5.0). For pairwise group comparisons, an unpaired t-test was applied when assumptions of normality and homogeneity of variances were met; otherwise, Welch’s or Mann-Whitney U tests were used as appropriate. Postoperative pain scores were analyzed using a mixed-effects model. A *p* value < 0.05 was considered to indicate statistical significance.

To evaluate factors influencing surgical duration, length of hospital stays, and postoperative pain, three separate multivariable linear regression models were constructed using the ordinary least squares (OLS) method in Python 3.10.18. The first model used operative time (in minutes) as the dependent variable, the second model analyzed hospital stay (in days), and the third model evaluated resting postoperative pain scores on the first day after surgery. All three models included the following independent variables: surgical procedure type, patient age, body mass index (BMI), number of pregnancies (gravida), number of deliveries (para), smoking status, presence of diabetes mellitus, malignancy status (benign vs. malignant) and the number of previous abdominal operations. The hospital stay and pain models additionally incorporated preoperative and postoperative hemoglobin levels as well as operative time. Categorical variables were processed using one-hot encoding, with Salpingo-oophorectomy defined as the reference category for procedure type. For each predictor, regression coefficients (β) and corresponding p-values were reported to quantify the magnitude and statistical significance of associations, adjusting for all other covariates.

## Results

All procedures were performed between December 2024 and September 2025 in the Department of Gynecology and Obstetrics at Greifswald University Hospital, a tertiary care center in Germany.

### Patient and surgical characteristics

The median age was 56.5 years (interquartile range: 43.25–68.75 years) and mean body mass index was 27.3 kg/m² ± 4.8 kg/m2 (range 16.9–37.5). Of the most common comorbidities observed, 24 patients (38.7%) had arterial hypertension, 8 patients (12.9%) had hypothyroidism and diabetes mellitus was present in 4 of all patients (6.5%). 15 patients (24.2%) had nicotine abuse. Prior abdominal surgeries were reported in 34 women (54.8%), including cesarean section in 5 patients (6.5%) and with 10 women (16.1%) having 2 or more surgical procedures done.

**Fig. 2:**
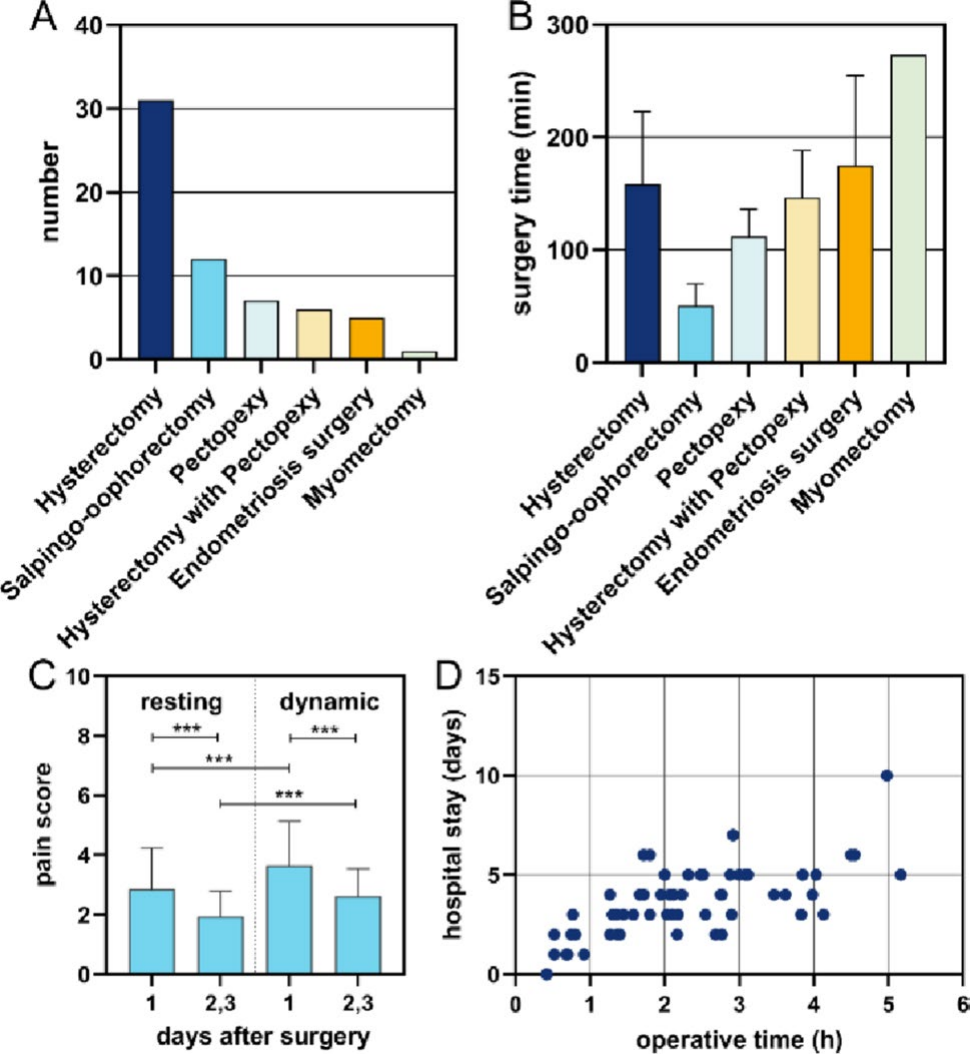
Surgical procedures, postoperative pain, and hospitalization duration. (**A**) The distribution of surgical procedures performed is shown. (**B**) Unadjusted operative durations are presented by procedure type. Procedure type was also the strongest predictor of operative duration in the multivariable linear regression model. (**C**) Resting and dynamic pain scores are illustrated for postoperative day 1 and days 2–3, showing a decline in reported pain over time. (**D**) A scatterplot of operative time versus hospital stay duration shows that longer operative times are significant associated with longer hospitalization days. in the multivariable model

Indications for surgery included both benign and malignant conditions in 46 and 16 women, respectively. We performed hysterectomy (*n* = 31), of which 8 cases underwent concurrent sentinel lymph node dissection using Indocyanine Green (ICG), and 2 underwent systemic pelvic and paraaortic lymph node dissection; hysterectomy with pectopexy (*n* = 6); pectopexy alone (*n* = 7); salpingo-oophorectomy (*n* = 12); myomectomy (*n* = 1); and endometriosis surgery (*n* = 5) (Fig. 2A). Patient characteristics and surgical variables are summarized in Table 1.

**Table 1:** Patient and surgical characteristics

Surgery Type	Hysterectomy	Salpingo-oophorectomy	Pectopexy	Endometriosis surgery	Hysterectomy with Pectopexy	Myomectomy
Count	31	12	7	5	6	1
Age [years]	53.5 ± 12.7	54.9 ± 10.9	69.6 ± 7.3	33.8 ± 6.1	72.3 ± 10.6	28
BMI [kg/m²]	27.6 ± 5.4	25.1 ± 3.8	26.7 ± 2.2	28.0 ± 6.6	27.7 ± 4.3	35
Hb-Difference [mmol/l]	-1.0 ± 0.6	-0.8 ± 0.3	-0.03 ± 0.5	-1.0 ± 0.6	-0.6 ± 0.4	-2,5
Op duration [min]	158.6 ± 64.3	50.6 ± 10.7	112.2 ± 24.3	175 ± 79.7	146.5 ± 42.0	273
Pain score; 1d resting [0–10]	3.2 ± 1.7	2.2 ± 0.8	2.3 ± 0.8	2.6 ± 1.1	2.7 ± 0.5	4.0
Pain score; 2,3d resting [0–10]	2.0 ± 0.65	1.5 ± 1.4	1.8 ± 0.4	2.8 ± 1.5	1.5 ± 0.5	3.0

The mean operation time was 136.2 ± 70.3 min (range 25–310). The mean docking time was 8.3 ± 2.2 min (range 3–13). In a multivariate linear regression model of surgical duration surgery type was the most significant predictor. After correction for age, BMI and comorbidities, Adnexectomy took 65.4 min (reference group, for age 56), hysterectomy 167.4 min (*p* ≤ 0.001), endometriosis surgery 192.5 min (*p* = 0.015), hysterectomy with pectopexy 184.8 min (*p* = 0.006), pectopexy 142.4 min (*p* = 0.03) and myomectomy 255.4 min (*p* = 0.007). Patient factors such as age, BMI, and comorbidities were not significantly associated with operative duration in this group (Fig. 2. B). The mean uterine weight in hysterectomy cases was 215.2 ± 237.5 g (range 27–930 g). The mean adnexal mass size after adnexectomy was 6.7 ± 2.6 cm (range 4–11 cm).

There was no post operative fever reported in all cases. No patients required postoperative blood transfusion. No patients developed incisional hernias following the operation. One patient experienced moderate postoperative vaginal bleeding approximately four hours after surgery. Another patient developed a subumbilical subcutaneous hematoma 3 days postoperatively. The mean hemoglobin, as a proxy for blood loss, changed significantly (paired t-test, *p* < 0.001), with − 0.93 ± 0.61 mmol/l (range − 2.8 to -0.1 mmol/l). The mean preoperative hemoglobin was 8.33 mmol/l, and the mean postoperative hemoglobin was 7.33mmol/l. There were no intraoperative complications or conversion to open laparotomy.

The mean pain score for resting patients on the first postoperative day was 2.9 ± 1.4, and 1.9 ± 0.9 on the second to third day, which were significantly lower than dynamic pain scores for the first postoperative day 3.7 ± 1.5 (mixed effects model, *p* < 0.0001) and the third postoperative day 2.6 ± 0.9 (mixed effects model, *p* < 0.000, Fig. 2. C). Among all variables, only the number of previous abdominal operations was significantly associated with increased postoperative pain scores (β = 0.81, *p* = 0.037). All other variables, including procedure type, hemoglobin levels, and operative time, showed no statistically significant association with postoperative pain in this model.

The mean hospital stay was 3.6 days ± 1.7 days (ranged between 0 and 9 days). In the multivariate regression analysis, only operative time (incision to closure) was independently associated with length of stay (+ 0.71 days per hour, *p* = 0.044, Fig. 2. D). While certain surgical types (e.g., hysterectomy with pectopexy or myomectomy) showed trends toward longer stays (+ 1.8 to + 2.1 days), these did not reach statistical significance.

## Discussion

Robotic surgery has transformed surgical practice over the years and represents a cutting-edge advancement in minimally invasive procedures, offering enhanced precision, improved instrument flexibility, and better ergonomic support for surgeons [5]. To optimize surgical outcomes beyond those achieved with multiport system, single-port surgery has been introduced, offering the potential for improved cosmesis, reduced postoperative discomfort, and greater patient satisfaction. The system is implemented at our clinic since December 2024. Many previous reports regarding single-port robotic surgery in gynecology, mainly Asian, state that surgical outcomes using the da Vinci SP are safe and equivalent to those of other systems [21, 22, 25, 26]. During the first nine months of utilizing the da Vinci SP system, we successfully performed 62 gynecological procedures. Surgical indications encompass both benign and malignant conditions. The spectrum of procedures ranges from simple salpingo-oophorectomies to complex surgeries for advanced endometriosis and oncological cases. Based on our initial experience, the da Vinci SP platform demonstrates robust versatility and efficacy, enabling the execution of the full range of minimally invasive gynecological surgeries without limitation.

### Intraoperative experience

Docking of the SP System seems to be less complicated and faster than previous robotic systems. Otherwise, it requires a shorter learning curve compared to the multi-port system. The mean docking time was 8.3 ± 2.2 min (range 3–13). Vizza et al. demonstrated a significant reduction (5-minute) in median docking time when using the da Vinci SP compared to MP [25]. Due to the lack of documented docking times for the first 32 patients, since documentation began at later stage, it was not possible to analyze the trend in docking time to assess the learning curve. Statistical analysis of the subsequent 32 patients showed no significant difference in docking times, indirectly suggesting that the surgeons achieved proficiency in system docking after approximately 30 procedures. Higuchi et al. performed a recent study to compare the surgical outcomes of hysterectomy using da Vinci SP and Xi und observed that the learning curve indicated proficiency after eight cases [27]. To further investigate these observations, a two-arm study comparing single-port with multi-port robotic surgery is currently planned at our institution.

The mean total operation time was 136.2 ± 70.3 min (range 25–310). Due to the use of a single port through a single incision for the entire procedure, most asymptomatic intra-abdominal peritoneal adhesions could be disregarded, provided they did not obstruct the surgical field of view. Such access is typically more complex with multi-port robotic systems, often resulting in longer operative times and a higher risk of complications due to the need for adhesiolysis. In a multivariate linear regression model evaluating surgical duration, the type of surgery emerged as the most significant predictor. Patient-related factors, including BMI, were not significantly associated with operative time in this cohort. Comparable results of total operating times have been published in previous reports [20, 28]. Park et al. reported that operative duration tended to be slightly longer with da Vinci SP in comparison to Xi, while the time needed for docking and wound incision was shorter [22].

The da Vinci SP system offers two different sizes of access ports (6 and 10 cm diameter). According to our initial experience, the larger port, particularly in obese patients, provides better instrument access to the abdomen and facilitates easier instrument replacement during surgery. However, in cases of subumbilical adhesions, inserting the larger port can be more challenging and increases the risk of clamping the omentum. Conversely, the smaller port is easier to insert and remove, especially in slim patients. Therefore, except in obese patients, we recommend the use of a small access port, particularly when subumbilical adhesions are anticipated. The access port of the da Vinci SP system includes two channels designed for assistant instruments: a 5–12 mm rotating access seal located on the access port cap and a 5–10 mm chamber seal on the side of the port chamber. These allow the assistant surgeon to help by performing tasks such as grasping, needle insertion and removal, tissue retraction, and suction. According to our experience, although the assistant surgeon’s working range through the access port channels is generally limited, it is adequate in almost all cases after the learning curve. In our series, we were able to perform pure single-port surgery in 58/62 (93,6%) of patients. In four cases, an additional 5 mm trocar was inserted on the left side of the abdomen. In two of these cases, additional access was required due to limited pelvic accessibility through the access port channels in obese patients. In the other two cases, the extra trocar was required by the assistant surgeon to create a peritoneal tent during the paraaortic lymph node dissection. Therefore, an additional assistant trocar should be considered during complex operative procedures or in obese patients.

Owing to the technical innovations of the da Vinci SP system, including multi-joint instruments, the interference commonly reported in single-site laparoscopic or robotic surgeries in previous studies has been reduced [20]. This represents a substantial improvement over earlier single-site techniques. However, the working range of the instruments at a single working spot and their articulation are still somewhat limited compared to the multi-port system, but after a learning curve, they are sufficient to perform gynecological procedures smoothly. If needed, the system’s relocating feature enables the surgeon to easily change the working spot and overcome the limited articulation of the instruments. Miyamura et al. noted that internal interference between the instruments within the abdominal cavity can probably still be a problem with the single-port system [26]. Based on our initial experience, the SP system appears particularly suitable for procedures in which intraoperative intraabdominal mobility plays an important role, such as pelvic and paraaortic lymph node dissection.

The camera of da Vinci SP is capable of performing COBRA action for even better visualization and less collisions with instruments. The current lack of advanced energy supported instruments like Vessel-Sealer and SynchroSeal limits the instrument choice for the surgeon. The Vessel Sealer and SynchroSeal enable reliable bipolar energy delivery for tissue fusion, vessel sealing, and dissection. The traction power of the instruments and the grasping strength of the needle driver are subjectively perceived as lower compared to the multi-port system. We are not the only surgical team to observe a subjective reduction in instrument strength; Misal et al. also report that achieving anterior or lateral traction can be particularly challenging when using the da Vinci SP system [23]. Therefore, removing bulky tissue, such as a large uterus or uterine myoma, may be easier with the multi-port system due to its superior instrument strength. The use of mono- or bipolar energy instruments during surgery produces intra-abdominal smoke, which can impair visualization in many cases. One minor difficulty we observe compared to multi-port systems is that, due to the higher positioning of the gas tubes connected to the access port, exchanging intra-abdominal gas to evacuate smoke is more challenging. When an additional trocar is inserted, gas evacuation should be performed through it without restrictions.

A key advantage of the da Vinci SP system is its ability to accommodate mild intraoperative patient repositioning, such as adjustments to the Trendelenburg position, without disconnecting the robot. In contrast, based on our experience, patient repositioning is technically unfeasible with multi-port systems unless the robot is re-docked. To the best of our knowledge, no published data currently exist regarding the feasibility or practicality of intraoperative repositioning with earlier multi-port robotic platforms.

Pelvic sentinel lymph node detection using ICG is bilaterally successful in all cases (8/8, 100%). However, the statistical significance of this result is limited due to the small number of patients who underwent sentinel lymph node dissection. The reported sensitivity of pelvic lymph node dissection using ICG in the literature ranges from 50 to 100% [29].

### Operative outcome

In our cohort, we observe no intraoperative complications or conversions to open laparotomy, and no postoperative fever, blood transfusions, or incisional hernias occur. One patient experienced postoperative vaginal bleeding following hysterectomy, which required operative management with transvaginal suturing of the vaginal cuff. Another patient developed a massive subcutaneous hematoma three days postoperatively, which was managed conservatively with local cooling therapy and showed gradual resolution. These complications are known risks associated with minimally invasive surgery and have been described in previous studies on the outcomes of robotic-assisted gynecological surgeries [11, 23, 27].

The mean hemoglobin changes significantly (paired t-test, *p* < 0.001), with − 0.93mmol/l ± 0.61 mmol/l (range − 2.8 to -0.1 mmol/l). Compared to previous studies reporting hemoglobin changes after minimally invasive surgery, no significant differences are observed between our results and those reported previously [27, 30–32]. Park et al. report a significant difference in the change of pre- and postoperative hemoglobin levels between different robotic systems in patients who underwent robotic hysterectomy, the da Vinci SP Group is showing a smaller change compared to the Xi system [22].

The mean resting pain score was 2.9 out of 10 on the first day and 1.9 on the second or third day. These scores are significantly lower than the corresponding dynamic pain scores, which were 3.7 on the first day and 2.6 on the third day. Among all variables, only the number of previous abdominal operations is significantly associated with increased postoperative pain scores (β = 0.81, *p* = 0.037). Patients with a higher number of prior surgeries report higher resting pain levels, resulting in a pain score increase of 0.81 per prior operation. The mean hospital stay was 3.6 days ± 1.7 days. In the multivariate regression analysis, only operative time is independently associated with length of stay (+ 0.71 days per hour, *p* = 0.044).

**Fig. 3:**
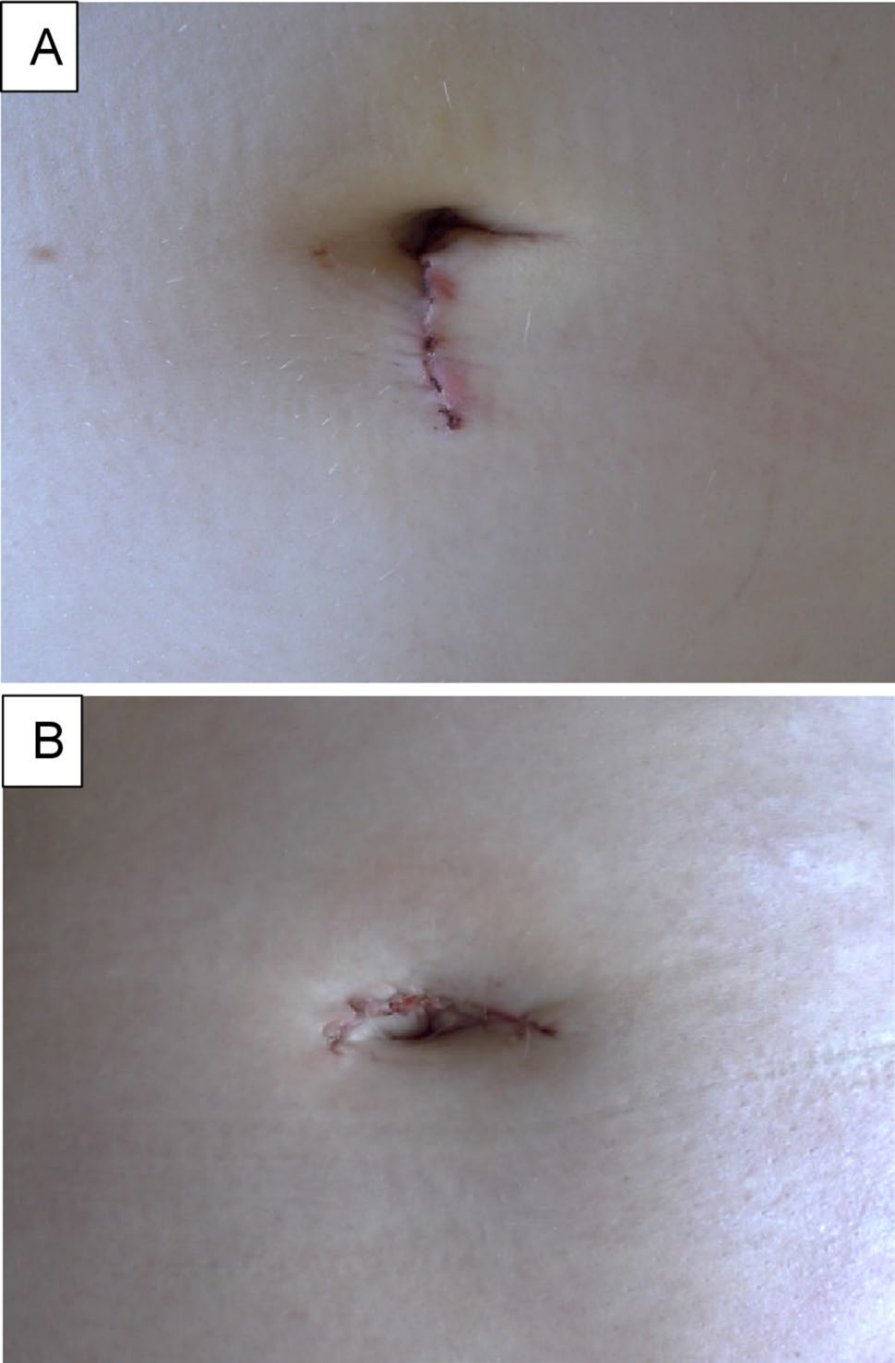
The postoperative umbilical scar on the 2nd day following single-port robotic surgery. (**A**) Vertical umbilical scar, (**B**) Horizontal umbilical scar

The skin incision in all patients was about 2.5–2.7 cm incision, with no intraoperative extension required in any case. In nearly all patients, wound healing and cosmetic outcomes are satisfactory (Fig. 3.), with the exception of one case with prolonged wound healing due to a postoperative wound hematoma, as previously described. In some patients, the scar is barely visible due to the deep umbilicus. Kwak et al. report that the umbilical wounds are similar to the ones they observed after single-port laparoscopic surgery [33]. Miyamura et al. and Song et al. noted an aesthetically better outcome after single incision surgeries compared to multi-port surgeries [26, 34].

This single-center study has certain limitations, including a limited sample size, its retrospective single-center design, heterogeneity in surgical indications and procedures, and a short follow-up period. Given the exploratory nature and modest sample size, we retained all consecutive cases to reflect the full clinical and procedural heterogeneity of single-port robotic gynecology. We adjusted for operative complexity using fixed effects for procedure class and malignancy; however, random-effects (multi-level) models could more fully capture clustering (e.g., by surgeon) in larger multi-center cohorts. However, these are outweighed by the study’s considerable strengths: as one of the earliest clinical series in Europe, and the first in German-speaking countries, to report on single-port robotic surgery across a range of gynecologic procedures, it provides additional valuable real-world evidence on safety and feasibility, introduces numerous previously undescribed tips and tricks, and highlights advantages and limitations not reported before.

## Conclusion

Despite the small sample size, the retrospective design of this study, and the heterogeneity of the operative group, the results appear promising. The use of the da Vinci single-port (SP) system in gynecology demonstrates good intraoperative performance and favorable postoperative outcomes.

Single-port robotic surgery in gynecological procedures may offer certain advantages over traditional multi-port systems. Operation time can be shorter due to easier docking and undocking, as well as a reduced need to remove asymptomatic intra-abdominal adhesions, particularly in the upper abdomen. The system’s ability to maneuver intra-abdominally with 360-degree movement enhances the intuitiveness of the robotic experience. Based on our initial experience, the da Vinci SP system could potentially complement existing multi-port systems in gynecologic surgeries. Additionally, aside from economic considerations, it might serve as an alternative to traditional laparoscopy in many cases in the future. However, single-port robotic surgery requires an additional learning curve beyond that of conventional endoscopic and robotic techniques. To validate these findings and evaluate long-term outcomes compared with multi-port systems, further prospective and controlled studies are warranted.

## Data Availability

All data used and analyzed during the current study are available from the corresponding author on request.
